# Facile Preparation of Oxygen-Vacancy-Engineered MoO_x_ Nanostructures for Photoreversible Switching Systems

**DOI:** 10.3390/nano11123192

**Published:** 2021-11-25

**Authors:** Hao Xu, Liangjing Zhang, Aiwu Wang, Juan Hou, Xuhong Guo

**Affiliations:** 1 Key Laboratory of Ecophysics, Department of Physics, College of Science, Shihezi University, Shihezi 832003, China; xu_hao88@sina.com; 2 Center for Advanced Material Diagnostic Technology, Shenzhen Technology University, Shenzhen 518118, China; zhangliangjing@sztu.edu.cn; 3 School of Chemical Engineering, East China University of Science and Technology, 130 Meilong Road, Shanghai 200237, China

**Keywords:** photochromic, MoO_x_ nanostructures, UV light, color switching

## Abstract

Photochromic materials have attracted increasing attention. Here, we report a novel photo-reversible color switching system based on oxygen-vacancy-engineered MoO_x_ nanostructures with water/N-methyl-2-pyrrolidone (NMP) as solvents. In this work, the system rapidly changed from colorless to blue under UV irradiation (360–400 nm) and slowly recovered its colorless state under visible light irradiation. The obtained oxygen vacancy-engineered MoO_x_ nanostructures exhibited good repeatability, chemical stability, and cycling stability. Upon UV light irradiation, H^+^ was intercalated into layered MoO_x_ nanostructures and the Mo^6+^ concentration in the H_x_MoO_x_ decreased, while the Mo^5+^ concentration increased and increased oxygen vacancies changed the color to blue. Then, it recovered its original color slowly without UV light irradiation. What is more, the system was highly sensitive to UV light even on cloudy days. Compared with other reported photochromic materials, the system in this study has the advantage of facile preparation and provides new insights for the development of photochromic materials without dyes.

## 1. Introduction

Materials that exhibit a change in their optical properties under the influence of either an electric field or light are known as discoloration materials. These materials are of interest in the realm of electrochromism and photochromism [1,2,3,4] and are of particular significance in novel technologies, such as sensors, pregnancy tests, electrochromic and photochromic displays, mirrors, and smart windows [5,6,7].

As an important semiconductor material, MoO_3_ shows strong localized surface plasmon resonance (LSPR) absorption, and the introduction of oxygen vacancies, aliovalent ions, and hydrogen ions induces a notable color change, thus making it an ideal photochromic material [8,9,10].

MoO_x_ (2 ≤ x ≤ 3) exists in different crystalline phases from MoO_2_ to MoO_3_. Pure MoO_3_ can involve different polymorphs, namely a thermodynamically stable orthorhombic phase (α-MoO_3_), hexagonal phase (h-MoO_3_), and metastable monoclinic crystal phase β-MoO_3_ (similar to ReO_3_ type structure). In fact, all reported MoO_x_ are basically constructed in different ways based on the construction of MoO_6_ octahedra, which exhibit layered structures, each layer connected by van der Waals force so that they can serve as hosts for dopant intercalants [11,12]. For example, MoO_3_ exhibiting a low oxygen vacancy concentration is white in color, whereas MoO_3_ with a high oxygen vacancy concentration is often blue or dark blue in color [13]. This phenomenon can be explained through the crystal rearrangements induced in the Mo−O units at the surface of the material during the reduction process, which results in a disordered surface structure containing many oxygen vacancies, thus imparting enhanced visible light absorption. The chromogenic response demonstrated by MoO_3_ demonstrates a stronger and more uniform absorption of visible light in its colored state and displays an increased open-circuit memory in comparison to the other transition-metal oxides [14]. MoO_3_ nanostructures of varying dimensions have been successfully prepared, including one-dimensional nanobelts and nanowires, alongside two-dimensional nanosheets [15,16,17,18,19]. However, only a limited number of studies have reported on the preparation of zero-dimension MoO_3_ quantum dots (QDs), especially with respect to their photochromic properties [20,21,22].

Top-down fabrication of layered nanostructures does not require harsh reaction conditions, thus low-cost high-yield preparation of materials is available. Furthermore, nanostructures prepared in this method do not develop an insulating ligand coating, which is detrimental to the electrical conductivity [23]. The ligand coating also exhibits a high defect concentration, which introduces many active edge sites for reactions [24]. The extent of the photochromism and electrochromism expressed by MoO_3_ is dominated by hydrogen radical intercalation or by electrochemical intercalation of lithium ions into the inter-layer gaps formed as a result of the van der Waal bonding. To date, ion intercalation has been proved to be the most effective method for increasing the free charge carrier concentrations in 2D materials [25,26].

Successful liquid phase exfoliation (LPE) of a layered material relies on overcoming the van der Waals forces between adjacent layers in the material [27,28,29]. This requires either an initial increase in the layer–layer spacing, which is induced through intercalation or by mixing with a solvent displaying equal surface tension properties [28,30]. During the exfoliation process, the efficiency of the exfoliation is strongly influenced by the extent of the similarity between the surface energy and Hansen solubility parameters (HSP) of the solvent and the target material [28,31,32]. As a result, using an appropriate exfoliation solvent can impart a significant change in the discoloration properties of MoO_3_. For example, a change of the solvent can affect the extent of the water photo-oxidation process and H^+^ ion intercalation in molybdenum oxide (MoO_x_) nanoflakes upon solar light illumination.

Herein, we propose a top-down synthesis method for the fabrication of layered nanostructures, using NMP/water as the exfoliation solvent, which is assisted by the hydrothermal method. Initially, bulk MoO_3_ is exfoliated to form 2D MoO_x_ nanoflakes in the NMP/water system, followed by high-temperature degradation into smaller particles. The photochromism of MoO_3_ can be briefly described as: in a MoO_3-x_ nanostructures sample of NMP/H_2_O mixed solvent, when ultraviolet light is irradiated on MoO_3_, the MoO_3_ on the surface is excited to separate electrons and holes, during which NMP acts as a good hole acceptor to help the separation of electrons and holes in this process, the separated holes react with surrounding water molecules, and H^+^ is produced in that way. The intercalation of H^+^ into MoO_3_ changed its crystal structure and reduced Mo^+6^, which resulted in the appearance of plasmon absorption peaks and the change of color. The H_x_MoO_3_ produced in the above process is generally considered to be an unstable metastable state. In the presence of oxygen, it tends to spontaneously dehydrate to form the corresponding oxide, thereby recovering the color. The as-obtained MoO_3-x_ nanostructures were characterized, and their optical and photochromic properties were evaluated. Particular samples displayed excellent photochromic performance and cycle stability. The influence of reaction time on nanostructures performance was also investigated in an attempt to validate their tunability through a slight adjustment in experimental parameters.

## 2. Materials and Methods

MoO_3_ powder (99%), ethanol (99.5%), and N-methyl-2-pyrrolidone (NMP, 98%) were obtained from Aladdin (Shanghai, China). Deionized water (18 MΩ) was used across all experimental procedures, and all reagents were used as received, without further purification.

Synthetic procedure:

MoO_3_ powder (0.03 g) was added into 10 mL of solvent containing deionized water and NMP in a 1:1 volume ratio (another sample used pure NMP as solvent). The mixture was then treated using sonication for 10 min, before being transferred into a 20 mL Teflon-lined stainless steel autoclave at 120 °C for 1, 3, 6, 12, 24, and 48 h to obtain samples exhibiting varying properties. Subsequently, each sample was placed in a centrifugal chamber at 12,000 rpm for 15 min and the supernatant was obtained from the samples. Finally, each sample was irradiated under a solar simulator for varying periods of time to obtain the MoO_3-x_ nanostructures. Residual powder from the samples could be collected and dried in a vacuum drying oven for reuse.

Characterization:

Transmission electron microscopy (TEM) images were taken using a JEOL JEM-2100 microscope at 200 kV (Tokyo, Japan). X-ray diffraction (XRD) patterns were recorded on a D/MAX2500 X-ray diffractometer (Tokyo, Japan) using Cu Kα radiation (λ = 1.5418 Å). The Fourier transform infrared-red (FTIR) spectroscopy was conducted on a Nicolet IS10 FT-IR spectrometer (Madison, WI, USA). X-ray photoelectron spectroscopy (XPS) was conducted on a Thermo Scientific Escalab 250 Xi X-ray photoelectron spectrometer (Waltham, MA, USA). The ultraviolet-visible absorption spectra were measured using a Thermo Evolution 220 UV-vis spectrometer (Waltham, MA, USA). The fluorescence spectra were measured using a HITACHI F-2700 fluorescence spectrophotometer (Tokyo, Japan). The source of the artificial UV light was MICROSOLAR300 Xenon lamp of Perfectlight (Beijing, China). The wavelength range was 320~780 nm. The power was 50 W, 19.6 W in visible region, 2.6 W in ultraviolet region. Dynamic light scattering (DLS) (ZEN3600, Malvern Instruments, Shanghai, China) was used to determine the size of nanoparticles. Raman spectra (Renishawplc, inVia) were recorded at 532 nm (Gloucestershire, England). Light transmittance was recorded by a FX2000 fiber spectrometer (Ideaoptics, Shanghai, China).

## 3. Results and Discussion

It is widely established that expanding the inter-layer spacing of layered materials through intercalation will weaken the van der Waals interactions between the layers. Previously published reports have revealed ion intercalation to be the most effective method for increasing the free charge carrier concentrations in 2D materials to induce plasmon resonance in the visible and near-infrared (NIR) regions [19]. Free electron concentration in MoO_x_ can be significantly increased by the introduction of oxygen vacancies through H^+^ ion intercalation into the inter-layers inherent to this material. Herein, intercalated H^+^ ions were used to induce an expansion of the inter-layer space, thereby increasing the free electron concentration through the introduction of oxygen vacancies [27].

**Figure 1:**
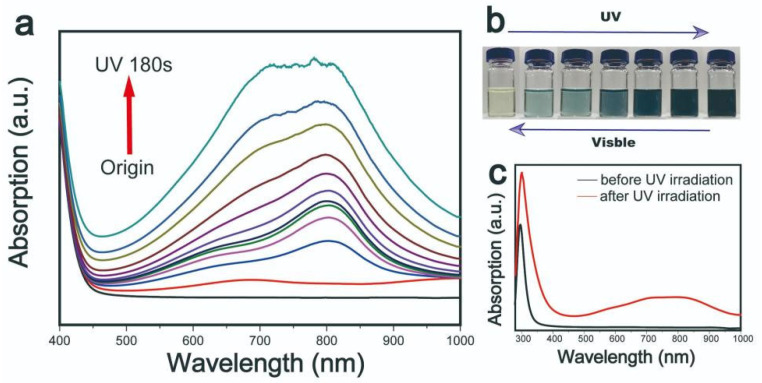
(**a**) The absorption spectra and (**b**) photos of MoO_3-x_ nanostructures irradiated by ultraviolet light for 180 s. (**c**) The absorption spectra of MoO_3-x_ nanostructures in the range of UV-Vis light.

N-methyl-2-pyrrolidone (NMP) was introduced as an exfoliation solvent. During the exfoliation process, the extent of the similarity between the HSP and the surface energy of the solvent and the target material strongly influences the efficiency of the exfoliation [19]. As a result, the choice of exfoliation solvent plays a considerable role in determining the plasmonic properties of MoO_x_ nanoparticles. Furthermore, a change of the solvent can affect the degree of the photo-oxidation of water and H^+^ ion intercalation in MoO_x_ nanoparticles upon solar light illumination. To characterize photochromic properties of MoO_x_ nanostructures, the samples were exposed to UV light for varying periods of time and their absorption response to the irradiation was recorded. The gathered visible absorption spectra underwent various changes, the results of which are provided in Figure 1.

In the case of the sample that was not exposed to UV light, the spectrum indicated that it exhibited a light yellow coloration, while there was no absorption peak observed in the visible light wavelength range. Upon exposure to UV light, the sample demonstrated a gradual darkening in color, the color change is reflected in its UV-visible absorption spectrum (Figure 1a), changing from light blue to dark blue to black (Figure 1b). As the exposure time increased, 600 nm broad absorption peaks gradually appeared in the <1000 nm range, while two further peaks were observed at approximately 700 and 800 nm. The appearance of the absorption peaks suggests that the MoO_3-x_ nanostructures possessed strong LSPR performance. MoO_3-x_ nanostructures had a strong absorption peak in UV range shown in Figure 1c.

The results of this study suggest that photo-excitation induces competing solvent and water oxidation processes, resulting in a reduction in H^+^ intercalation. NMP forms an aprotic solvent, in which the molecules of the solvent are not hydrogen bonded to one another, meanwhile the photo-oxidation of NMP is weak. Therefore, in the NMP/H_2_O system, water is photo-oxidized under ultraviolet light instead of NMP and H^+^ produced in this process is intercalated into MoO_3_. On the other hand, NMP is provided with the highest proton affinity (∼920 kJ mol^−1^) [20] and lowest proton dissociation rate, which induces a higher degree of H^+^ intercalation in NMP in comparison to other solvents [27].

The mechanism of ultraviolet (UV) light irradiation is summarized below [33], * represent the excited state:

MoO_3_ + hv → MoO_3_* + h^+^ + e^−^ (1)

2h^+^ + H_2_O → 2H^+^ + 1/2O_2_ (2)

MoO_3_ + XH^+^ +Xe^−^ → H_X_MoO_3-x_ (3)

Throughout the photo-oxidation process, H_2_O provides a source of hydrogen ions, while NMP performs the role of a hole trapping acceptor, which can promote the separation of electrons and holes, thus speeding up the reaction process. This promotion induces an increase of migration of hydrogen ions in the system.

**Figure 2:**
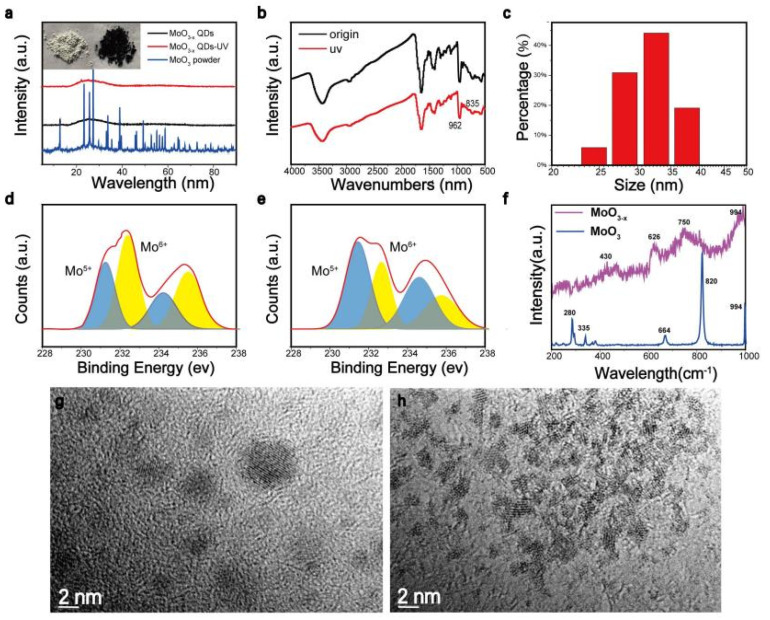
(**a**) XRD pattern of MoO_3-x_ nanostructures and photographs of α-MoO_3_ powder and MoO_3-x_ nanostructures powder (inset image). (**b**) FTIR spectra of MoO_3-x_ nanostructures with and without UV. (**c**) DLS spectrum of MoO_3-x_ nanostructures. (**d**) Mo 3d core level spectra of MoO_3-x_ nanostructures without UV excitation and (**e**) Mo 3d core level spectra of MoO_3-x_ nanostructures without UV excitation. (**f**) Raman spectra of MoO_3_ and MoO_3-x_ nanostructure. (**g**) TEM images of MoO_3-x_ nanostructures without ultraviolet light irradiation and (**h**) irradiated by ultraviolet light.

Small particles, including nanostructures, were detached from the rough MoO_3_ particles, as shown in Figure 2. To further explore the structure characteristics, the XRD (Figure 2a) was carried out to obtain the crystalline phase of MoO_x_ nanostructures. Sharp diffraction peaks located in the range of 20°–30°, representing α-MoO_3_, became wide in MoO_x_ nanostructures, indicating that an amorphous structure was formed. In Figure 2b, IR bands occurring in the range of 962 cm^−1^ to 835 cm^−^^1^ were Mo=O bond stretching vibration and Mo-O-Mo bond stretching vibration, which prove that the obtained nanostructure was molybdenum oxide [20]. DLS in Figure 2c shows the nanostructures had an average size of 30 nm, which contradicted most of our TEM images—the increase of statistical size could be attributed to the aggregation of small particles. In Figure 2d,e, the core level spectra of Mo3d in H_x_MoO_3_ nanostructures were introduced. The double peaks at 235.6 eV and 232.2 eV corresponded to the binding energy of 3d5/2 and 3d3/2 orbital electrons of Mo^6+^, while 234.3 eV and the double peaks at 231.2 eV represented the binding energy of the 3d5/2 and 3d3/2 orbital electrons of Mo^5+^. It is obvious that after ultraviolet light irradiation, the Mo^6+^ content in the H_x_MoO_3_ nanostructures decreased and the Mo^5+^ content increased, which indicates that the nanostructures underwent surface structure reconstruction, and the concentration of oxygen vacancies increased, accompanied by a decrease in the valence state of molybdenum.

The Raman spectra of the MoO_3-x_ structure were also investigated (Figure 2f) before and after illumination, the 280 cm^−1^ represented the wagging mode of the double bond O=Mo=O, the peaks at 664 cm^−1^ and 820 cm^−1^ could be assigned to the stretching modes of the triple coordinated oxygen (Mo_3_-O), and the doubly coordinated oxygen (Mo_2_-O), respectively, the 335 cm^−1^ was assigned to the Mo_3_-O bending mode. For the samples of MoO_3-x_ structure after illumination, traces of MoO_3-x_ were also identified by two additional peaks 430 cm^−1^ and 750 cm^−1^ due to the modification of original Mo_2_-O bond [33]. From Raman analysis, the existence of some substoichiometry in the MoO_3-x_ structure can be possibly due to the intercalation of H^+^ from the solvent and increase of oxygen vacancy in the presence of significant heat and force generated during the solvothermal process [19,20,33,34]. After centrifugation, the resultant MoO_3-x_ nanostructures were observed using transmission electron microscopy (TEM). The TEM images of the MoO_3-x_ nanostructures are illustrated in Figure 2g,h, revealing an average diameter of approximately several nanometers.

**Figure 3:**
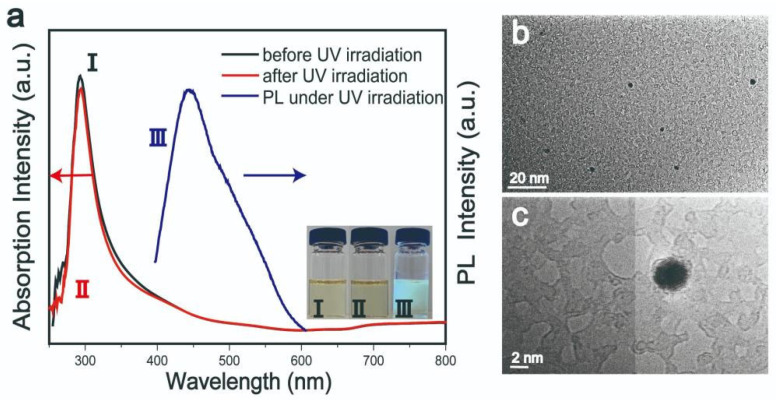
(**a**) UV-visible absorption spectra, photoluminescent spectra, and photographs before (I), during (III), and after UV irradiation (II). (**b**,**c**) TEM images of MoO_3_ QDs with pure NMP as solvent.

Concerning samples using pure NMP as solvent, these exhibited quite different optical behavior (Figure 3a). The nanostructures had fluorescent peaks at 450 nm under 360 nm excitation light. In addition, the absorption peaks in the range of 700–900 nm had no response to irradiation, while at the same time, the color of the samples was constant. Figure 3b,c are TEM images of samples using pure NMP as solvent. This behavior may be attributed to the different formation mechanisms of nanostructures in water/NMP and pure NMP solvents. In the water/NMP mixed solvent, water may have combined with holes generated through light excitation to produce H^+^, which is related to the H^+^ intercalation mechanism. As there was no H^+^ production in the NMP solvent, the photo-corrosion mechanism explains the behavior of this system more accurately. The oxidation strength of NMP is limited, and the photo-generated holes cannot be consumed quickly. Owing to their strong oxidizing ability, vacancy accumulation after continuous light excitation may corrode the lattice structure of MoO_3_. This is known as photo-corrosion and most often occurs in semiconductor materials used in light-related applications [20,27].

In this study, the number of oxygen vacancies present in the MoO_3-x_ nanostructures was influenced by the length of the reaction time. Herein, we prepared a series of MoO_3-x_ nanostructures exhibiting varying photochromic behaviors to analyze and compare their UV-visible absorption spectra.

**Figure 4:**
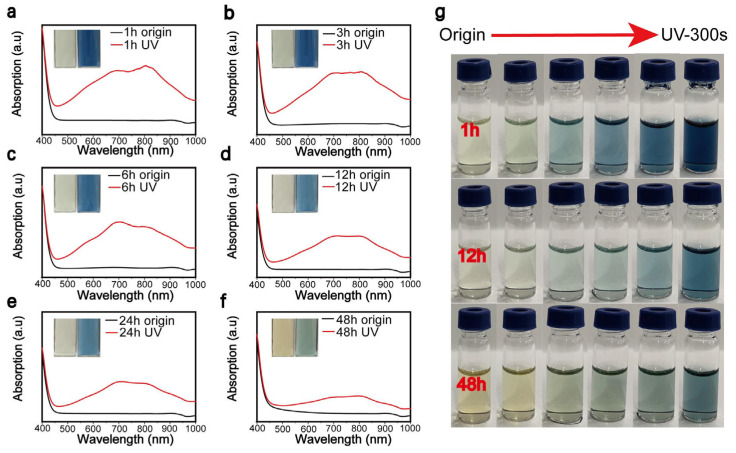
(**a**–**f**) The absorption spectrum for the MoO_3-x_ nanostructure underwent varying reaction times of 1, 3, 6, 12, 24, and 48 h, and origin samples were irradiated for 5 min by Xenon lamp to create UV samples. (**g**) Photographs of MoO_3-x_ nanostructures irradiated by Xenon lamp for 300 s.

Figure 4a–f are the UV-visible absorption spectra generated by the MoO_3-x_ nanostructure samples, which underwent varying reaction times of 1, 3, 6, 12, 24, and 48 h. Each sample underwent UV irradiation for 5 min, which facilitated an accurate analysis of the absorption effects demonstrated in the sample solely as a function of the reaction time. As the reaction time increased, a gradual reduction in the peak intensity was confirmed; that is, the sensitivity to UV light decreased. A photograph of the color gradation exhibited by the MoO_3-x_ nanostructures was performed in Figure 4g, samples underwent the reaction for 1, 12, and 48 h before UV irradiation for 300 s.

We speculated that the reaction proceeded rapidly under hydrothermal conditions. Upon completion of the intercalation reaction, and as the reaction time increased, NMP played a significant role in controlling the size and morphology of the nanostructures. In this case, the absorption energy was greater than the forbidden band gap. Since the generated electron–hole pairs could not be completely consumed, the highly oxidizing holes oxidized the crystal of MoO_3_, thus disrupting the lattice structure.

As oxygen exhibits a significant larger electronegativity than hydrogen, the majority of binary metal oxides show higher stability than their corresponding hydrides [30,35]. The hydrogenation process in this article was used to facilitate molybdenum oxide to form unstable H_x_MoO_3_ by ultraviolet light irradiation. This switching of the crystal structure induced a change in the optical and electrical properties of the material. The original α-MoO_3_ was a semiconductor with a band gap of 3.2 eV. The charge induced upon irradiation was transferred from the newly formed Mo^5+^ state to the adjacent Mo^6+^ state, and H_X_MoO_3_ appeared blue. H_X_MoO_3_ adopts a metastable state and will spontaneously be oxidized to the corresponding oxide [36]. Under high temperatures, it can accelerate the spontaneous reduction process [37].

The MoO_3-x_ nanostructure samples prepared in this study were light yellow in color prior to UV light irradiation. Upon irradiation by sunlight or UV light, the structure of the MoO_3-x_ nanostructures changed in accordance with the element valence state, and a corresponding color change at the surface was observed. From the initial light yellow color, it turned light blue, before changing to dark blue through to black, where LSPR absorption peaks appeared in the visible light wavelength range of the absorption spectrum. This was a reversible process. When an irradiated sample was placed in an environment without UV light, its color returned to its initial light yellow slowly. Temperature imparts a considerable effect on the color change recovery process. When the dark sample was heated to 80 °C, the recovery rate increased, and the total recovery time was significantly reduced to the order of minutes.

**Figure 5:**
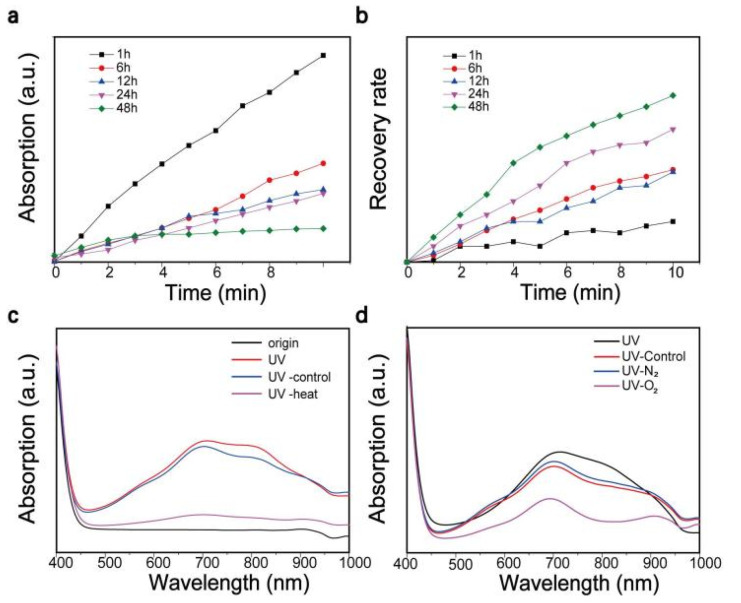
(**a**) The absorption peaks of MoO_3-x_ nanostructures under UV 10 min of UV irradiation using a Xenon lamp, samples were obtained at different reaction times. (**b**) The self-recovery rate of MoO_3-x_ nanostructures obtained at different reaction times after 10 min of UV irradiation using a Xenon lamp. The recovery rate is defined as the ratio of the amount of absorption intensity recovered to the absorption intensity before recovery. (**c**) The effect of heating on photochromism of MoO_3-x_ nanostructures. Origin sample was irradiated for 10 min to create a UV sample, then the UV sample was divided to two parts, treated with and without 20 min heating, marked as UV-heating and UV-control. (**d**) The effect of oxygen on photochromism of MoO_3-x_ nanostructures—the UV sample was obtained via irradiation for 10 min and samples treated with O_2_, N_2_, and air were marked as UV-O_2_, UV-N_2_, and UV-control, respectively.

To explore the effect of synthesis time on the photochromic behavior of MoO_3-x_ nanostructures, the reaction temperature was set at 120 °C and MoO_3-x_ nanostructures subjected to various reaction times were obtained. Their photochromic properties were analyzed by UV-visible absorption spectroscopy. The various samples were subjected to the same experimental conditions and the discoloration–recovery line chart is illustrated in Figure 5a,b. It could be concluded from Figure 5a that, as the reaction time increased, the UV-visible absorption peak intensity reduced and the sample became less sensitive to UV light. As previously discussed, this may be attributable to the different formation mechanisms exhibited by nanostructures formed in mixed water/NMP and single NMP solvent systems [20,27,38]. The trend was completely reversed when we investigated the color restoration of MoO_3-x_ nanostructure samples (Figure 5b). Samples prepared under a long reaction time formed a H_x_MoO_3_ metastable structure and brought about an increased rate of dehydration and reduction. This is attributed to the smaller ion size and greater number of surface sites displayed by nanostructures obtained under a longer reaction time, which are beneficial for embedding, separating hydrogen, and decomposing metastable H_x_MoO_3_ [36,37]. Some factors that impacted the recovery process, such as temperature and oxygen, are shown in Figure 5c,d. Briefly, high temperature and oxygen can accelerate the recovery process. During that process, the H_x_MoO_3_ with oxygen vacancy is oxidized and hydrogen atoms are taken away by surrounding oxygen, accompanied by a reduction in the oxygen vacancy [34,39].

**Figure 6:**
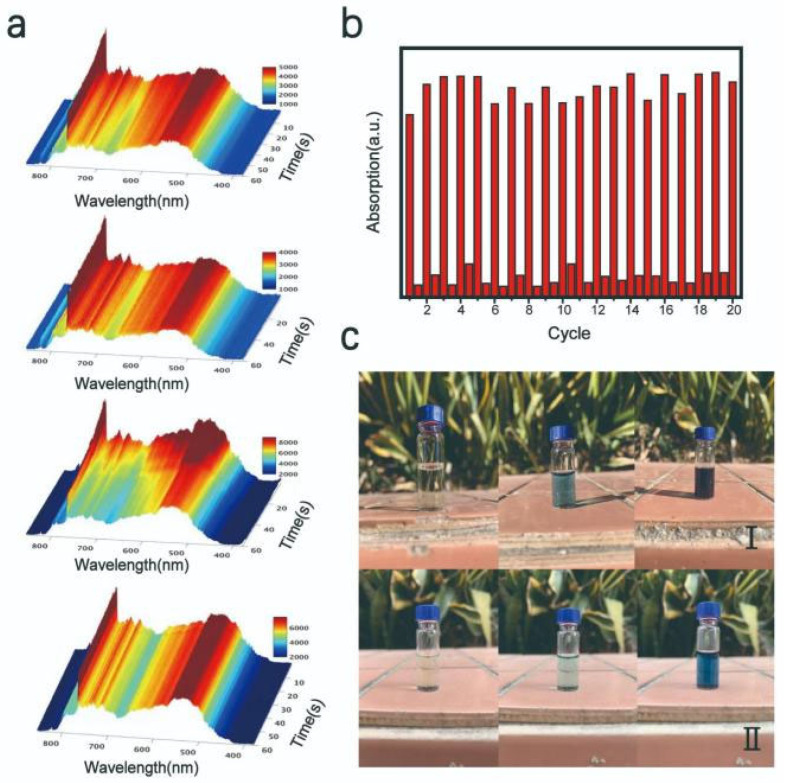
(**a**) Spectra of the Fiber Optic spectrometer. (**b**) Cycle stability test. (**c**) Discoloration performance of MoO_3-x_ nanostructures in sunny weather (I) and cloudy weather (II).

In Figure 6a, a fiber optic spectrometer was used to evaluate and characterize the light transmittance of the nanostructures. The variation in the light transmittance was recorded in 100 ms intervals throughout the test. It is clear that the light intensity decreased gradually as the UV illumination time increased from 0 to 60 s in the 700–800 nm wavelength range. This suggests that the light absorption capacity of the sample increased in this wavelength band, which corresponded to an increase in the peak value recorded in UV-visible absorption spectrum. The cycle performance test was used to characterize the photochromic stability of MoO_3-x_ nanostructures. The obtained MoO_3-x_ nanostructure solution was irradiated under a solar simulator for 5 min. The UV-visible absorption spectrum was recorded at this point, and the sample was then stored in an environment without UV light. Upon completion of the recovery period, the UV-visible absorption spectrum was recorded again. The test was repeated for 20 times, as shown in Figure 6b. Photographs of samples after exposure to bright sunlight, as well as also cloudy conditions, for 0, 5, and 15 min were used as controls (Figure 6c). It was observed that the MoO_3-x_ nanostructure samples exhibited a strong discoloration effect, even when placed in an environment of low UV irradiation conditions, demonstrating that this material provides considerable potential for practical applications.

## 4. Conclusions

In summary, oxygen-vacancy-engineered MoO_x_ nanostructures were fabricated by a modified top-down method combining intercalation and the solvent–thermal method. In the case of the mixed solvent system of water/NMP, water was introduced to produce H^+^ through photo-oxidation to facilitate intercalation into MoO_x_ exhibiting a layered structure. An increase in proton intercalation and oxygen vacancy in molybdenum oxide nanostructures resulted in a significant increase in their free electron concentrations and changes in their substoichiometry displayed intense LSPR in the visible light region. In the presence of oxygen, the hydrogen atoms were taken away combined with oxygen, and metastable H_X_MoO_3_ were oxidized, accompanied by a reduction of LSPR and color restoration. A series of MoO_x_ nanostructures exhibiting consistent photochromic behavior were prepared through control of the reaction time and temperature. The synthesized samples exhibited excellent photochromic properties and cycle stability. In this context, our oxygen-vacancy-engineered MoO_x_ nanostructures provide an important stimulus to the development of practical photochromic materials

## 5. Patents

There is a Chinese patent 202110009536.X resulting from the work reported in this manuscript.

## Data Availability

Data is available on the request from the corresponding author.
